# Enhanced Lignin Monomer Production Caused by Cinnamic Acid and Its Hydroxylated Derivatives Inhibits Soybean Root Growth

**DOI:** 10.1371/journal.pone.0080542

**Published:** 2013-12-03

**Authors:** Rogério Barbosa Lima, Victor Hugo Salvador, Wanderley Dantas dos Santos, Gisele Adriana Bubna, Aline Finger-Teixeira, Anderson Ricardo Soares, Rogério Marchiosi, Maria de Lourdes Lucio Ferrarese, Osvaldo Ferrarese-Filho

**Affiliations:** Laboratory of Plant Biochemistry, University of Maringá, Maringá, Paraná, Brazil; The Ohio State University, United States of America

## Abstract

Cinnamic acid and its hydroxylated derivatives (*p*-coumaric, caffeic, ferulic and sinapic acids) are known allelochemicals that affect the seed germination and root growth of many plant species. Recent studies have indicated that the reduction of root growth by these allelochemicals is associated with premature cell wall lignification. We hypothesized that an influx of these compounds into the phenylpropanoid pathway increases the lignin monomer content and reduces the root growth. To confirm this hypothesis, we evaluated the effects of cinnamic, *p*-coumaric, caffeic, ferulic and sinapic acids on soybean root growth, lignin and the composition of *p*-hydroxyphenyl (H), guaiacyl (G) and syringyl (S) monomers. To this end, three-day-old seedlings were cultivated in nutrient solution with or without allelochemical (or selective enzymatic inhibitors of the phenylpropanoid pathway) in a growth chamber for 24 h. In general, the results showed that 1) cinnamic, *p*-coumaric, caffeic and ferulic acids reduced root growth and increased lignin content; 2) cinnamic and *p*-coumaric acids increased *p*-hydroxyphenyl (H) monomer content, whereas *p*-coumaric, caffeic and ferulic acids increased guaiacyl (G) content, and sinapic acid increased sinapyl (S) content; 3) when applied in conjunction with piperonylic acid (PIP, an inhibitor of the cinnamate 4-hydroxylase, C4H), cinnamic acid reduced H, G and S contents; and 4) when applied in conjunction with 3,4-(methylenedioxy)cinnamic acid (MDCA, an inhibitor of the 4-coumarate:CoA ligase, 4CL), *p*-coumaric acid reduced H, G and S contents, whereas caffeic, ferulic and sinapic acids reduced G and S contents. These results confirm our hypothesis that exogenously applied allelochemicals are channeled into the phenylpropanoid pathway causing excessive production of lignin and its main monomers. By consequence, an enhanced stiffening of the cell wall restricts soybean root growth.

## Introduction

Higher plants regularly release secondary metabolites into the soil environment that can influence the growth and development of neighboring plants both positively and negatively. This is known as allelopathy, defined as both the ability of plants to protect themselves using natural allelochemicals and the chemical communication between microbe–microbe, plant–microbe, plant–insect or plant–herbivore [1]. A large number of allelochemicals are commonly found in soils at concentrations between 0.01 and 0.1 mM and affect plant growth at concentrations of up to 10 mM [2], [3]. Allelochemicals typically suppress seed germination, causing disorders of root growth and inhibiting plant growth. Moreover, they alter several physiological and biochemical processes such as water utilization, mineral uptake, photosynthesis, amino acid metabolism, protein synthesis, glycolysis, mitochondrial respiration and ATP synthesis, among countless others [1].

Root growth is characterized by high metabolic activity; therefore, the roots are considered to be quite susceptible to the stress induced by allelochemicals in the soil solution. After uptake, the walls of root cells are directly exposed to an excess of allelochemicals. Lignification, the metabolic process of sealing a plant cell wall by lignin deposition, occurs during the course of normal tissue development and is an important step during root growth. Lignin is a polymer formed by the oxidative coupling of *p*-hydroxycinnamyl alcohol monomers (called monolignols), which are products of the phenylpropanoid pathway [4]. In the first steps of this metabolic pathway, the deamination of l-phenylalanine by phenylalanine ammonia-lyase (PAL) produces *t*-cinnamate. This step is followed by hydroxylation of the aromatic ring, catalyzed by cinnamate 4-hydroxylase (C4H), to generate *p*-coumarate. The next step is the activation of the acid to a thioester via 4-coumarate:CoA ligase (4CL) to yield *p*-coumaroyl-CoA. Subsequently, *p*-coumaroyl-CoA is transesterified to shikimic/quinic acid ester derivatives by the action of 4-hydroxycinnamoyl-CoA:shikimate/quinate 4-hydroxycinnamoyltransferase (HCT). A hydroxylation reaction of *p*-coumaroyl-CoA shikimate/quinate produces caffeoyl-CoA, which is then transesterified again by HCT to generate caffeoyl-CoA. A methoxylation reaction catalyzed by caffeoyl-CoA *o*-methyltransferase (CCoAOMT) produces feruloyl-CoA from caffeoyl-CoA. By the sequential action of cinnamoyl-CoA reductase (CCR), ferulate 5-hydroxylase (F5H), caffeic acid 3-*o*-methyltransferase (COMT) and cinnamyl alcohol dehydrogenase (CAD), the CoA thioesters are converted into monolignols (*p*-coumaryl, coniferyl and sinapyl alcohols). Finally, the oxidative polymerization of these three monolignols, via the action of peroxidases (POD), generates the *p*-hydroxyphenyl (H), guaiacyl (G) and syringyl (S) units of the lignin polymer [4].

Lignin plays a crucial role in the resistance to biotic and abiotic stresses [5], [6]. Correlations between plant growth reduction and increased lignin content in roots have been considered as a defense against allelochemical and other stresses [7]–[11]. Because of these stresses, lignification can limit cell expansion and the capacity for nutrient uptake, and thus the ability to sustain plant growth. Previously, we demonstrated that hydroxycinnamic derivatives such as ferulic, *p*-coumaric and caffeic acids impaired soybean root growth associated with changes in phenylpropanoid pathway enzymes and premature cell wall lignification [12]–[14]. Based on these reports, we hypothesized that these allelochemicals can be directly channeled into the phenylpropanoid pathway, increasing the amount of lignin monomers, solidifying the cell wall and reducing root growth. Because a lignin polymer is mainly formed from H, G and S units, we hypothesized that changes in this monomeric composition could be one mode of action of these allelochemicals. To confirm this hypothesis, we examined the effects of cinnamic acid and its hydroxylated derivatives (*p*- coumaric, caffeic, ferulic and sinapic acids) on soybean root growth, total lignin and the composition of H, G and S monomers. To evaluate the possible entry of exogenous allelochemicals into the phenylpropanoid metabolic route, we performed experiments using three selective enzymatic inhibitors of this pathway: 2-aminoindan-2-phosphonic acid (AIP, a competitive inhibitor of the enzyme PAL), piperonylic acid (PIP, a *quasi*-irreversible inhibitor of the enzyme C4H) and 3,4-(methylenedioxy)cinnamic acid (MDCA, a competitive inhibitor of the enzyme 4CL).

## Materials and Methods

### General procedures

Soybean (*Glycine max* L. Merrill) seeds were surface-sterilized with 2% sodium hypochlorite for 2 min, rinsed extensively with deionized water and germinated in the dark (at 25°C) on two sheets of moistened filter paper. A total of 25 three-day-old seedlings of uniform size were supported by an adjustable acrylic plate and dipped into a glass container (10×16 cm) filled with 200 mL of half-strength Hoagland's solution (pH 6.0) with or without 1.0 mM cinnamic, *p*-coumaric, caffeic, ferulic or sinapic acids. All nutrient solutions were buffered with 17 mM potassium phosphate buffer to eliminate the effects of a very low pH. The concentration of each allelochemical was selected based on a survey of the literature [2], [3], [15]–[17]. Additional experiments with 10 µM AIP, 0.01 mM PIP or 2 mM MDCA were conducted as indicated in the figure legends. The containers were maintained in a growth chamber at 25°C with a light/dark photoperiod of 12/12 h and a photon flux density of 280 µmol m^−2^ s^−1^ for 24 h. The roots were measured before incubation and at the end of the experiments, and differences in length were calculated for all the samples. Where indicated, the fresh root weight was determined immediately after incubation. The dry weight was estimated after oven-drying the samples at 80°C until they reached a constant weight. Cinnamic, *p*-coumaric, caffeic, ferulic and sinapic acids as well as PIP and MDCA were purchased from Sigma–Aldrich (St Louis, MO, USA), and AIP was generously donated by Jerzi Zón (Wroclaw University of Technology, Poland). All other reagents were of the purest grade available or of chromatographic grade.

### Quantification of lignin and monomer composition

After the incubation period, dry roots (0.3 g) were homogenized in 50 mM potassium phosphate buffer (7 mL, pH 7.0) with a mortar and pestle and transferred into a centrifuge tube [9]. The pellet was centrifuged (1,400×g, 4 min) and washed by successive stirring and centrifugation as follows: twice with phosphate buffer (pH 7.0) (7 mL), 3× with 1% (v/v) Triton® X-100 in pH 7.0 buffer (7 mL), 2× with 1 M NaCl in pH 7.0 buffer (7 mL), 2× with distilled water (7 mL), and 2× with acetone (5 mL). The pellet was dried in an oven (60°C, 24 h) and cooled in a vacuum desiccator. The dry matter obtained was defined as a protein-free cell wall fraction. Further, all dry protein-free tissue was placed into a screw-cap centrifuge tube containing the reaction mixture (1.2 mL of thioglycolic acid plus 6 mL of 2 M HCl) and heated (95°C, 4 h). After cooling at room temperature, the sample was centrifuged (1,400×g, 5 min), and the supernatant was discarded. The pellet contained the complex lignin–thioglycolic acid (LTGA). The pellet was washed 3× with distilled water (7 mL), and the LTGA was extracted by shaking (30°C, 18 h, 115 oscillations min^−1^) in 0.5 M NaOH (3 mL). After centrifugation (1,400×g, 5 min), the supernatant was stored. The pellet was washed again with 0.5 M NaOH (3 mL) and mixed with the supernatant obtained earlier. The combined alkali extracts were then acidified with concentrated HCl (1.8 mL). After precipitation (0°C, 4 h), LTGA was recovered by centrifugation (1,400×g, 5 min) and washed 2× with distilled water (7 mL). The pellet was dried at 60°C, dissolved in 0.5 M NaOH, and diluted to yield an appropriate absorbance for spectrophotometric determination at 280 nm. The lignin content is expressed as mg LTGA g^−1^ dry weight.

The oxidation of alkaline nitrobenzene was used to determine the lignin monomer composition [13]. The protein-free cell wall fraction (50 mg) obtained above was sealed in a Pyrex® ampule containing 1 mL of 2 M NaOH and 1 mL of nitrobenzene prior to heating at 170°C for 90 min with occasional shaking during the reaction. The sample was then cooled at room temperature, washed twice with chloroform, acidified to pH 2.0 with 2 M HCl and extracted twice with chloroform. The organic extracts were combined, dried and resuspended in 1 mL of a mixture of methanol and 4% acetic acid in water (20∶80, v/v). All the samples were filtered through a 0.45-µm disposable syringe filter (Hamilton® Co., Nevada, USA), and 20 µL aliquots were analyzed in an high performance liquid chromatography (HPLC) system (Shimadzu® 10AVP, Tokyo, Japan) equipped with an LC-10AD pump, a Rheodyne® injector, an SPD-10A UV detector, a CBM-101 Communications Bus Module, and a Class-CR10 workstation system. A reversed-phase Shimpack® CLC-ODS column (150 mm×4.6 mm, 5 µm), protected with an equivalent pre-column, was used at 30°C. The mobile phase consisted of a mixture of methanol and 4% acetic acid in water (20∶80, v/v) with a flow rate of 1.2 mL min^−1^ for an isocratic run of 20 min. Quantification of the monomer aldehyde products (*p*-hydroxybenzaldehyde, vanillin and syringaldehyde) released by nitrobenzene oxidation was performed at 290 nm using the corresponding standards. The results are expressed as mg monomer g^−1^ cell wall.

### Statistical analysis

The experimental design was completely randomized, and each plot was represented by one glass container with 25 seedlings. The data were expressed as the mean of four independent experiments ± SE. To determine significant differences, an analysis of variance was performed with the statistical programs Prism® (GraphPad 5.0, USA) and Sisvar® (UFLA, Brazil). The differences between the parameters were evaluated using Dunnett's multiple comparison test (Prism® software) and the Scott–Knott test (Sisvar® software). *P* values≤0.05 were considered statistically significant.

## Results

### Effects of allelochemicals on root growth

Table 1 summarizes the effects of allelochemicals on soybean root growth. Cinnamic acid was the most effective inhibitor of root length, causing a 93.3% reduction compared with the control. *p*- Coumaric, caffeic and ferulic acids reduced root growth by 43.3%, 34.2% and 41.7%, respectively. The effects of the allelochemicals were also evident for root fresh weight, which significantly decreased after cinnamic (26.6%), *p*- coumaric (13.9%), caffeic (20.1%) and ferulic (16.9%) acid treatments when compared with the control. Similarly, the root dry weights were reduced by cinnamic (25.0%), *p*- coumaric (6.3%), caffeic (18.7%) and ferulic (12.5%) acids.

**Table 1:** Changes in the root length, root fresh weight and root dry weight of soybean seedlings treated for 24(CIN), *p*-coumaric (*p*-COU), caffeic (CAF), ferulic (FER) and sinapic (SIN) acids.

Condition	Root length (cm)	%	Fresh weight (g)	%	Dry weight (g)	%
**Control**	2.54±0.04		2.44±0.04		0.16±0.005	
**CIN**	0.17±0.02^*^	−93.3	1.79±0.05^*^	−26.6	0.12±0.003^*^	−25.0
***p*** **-COU**	1.44±0.05^*^	−43.3	2.10±0.04^*^	−13.9	0.15±0.003^*^	−6.3
**CAF**	1.67±0.01^*^	−34.2	1.95±0.01^*^	−20.1	0.13±0.009^*^	−18.7
**FER**	1.48±0.04^*^	−41.7	2.03±0.02^*^	−16.8	0.14±0.003^*^	−12.5
**SIN**	2.43±0.03^ns^		2.46±0.02^ns^		0.16±0.001^ns^	

Values (*N* = 4 ± SE) significantly smaller than the control (*P*≤0.05, Dunnett's multiple comparison test) are marked with an asterisk (*). ns = not significant. The % symbol represents inhibition of statistically significant means in comparison with the control (0 mM).

### Effects of allelochemicals on lignin content

**Figure 1:**
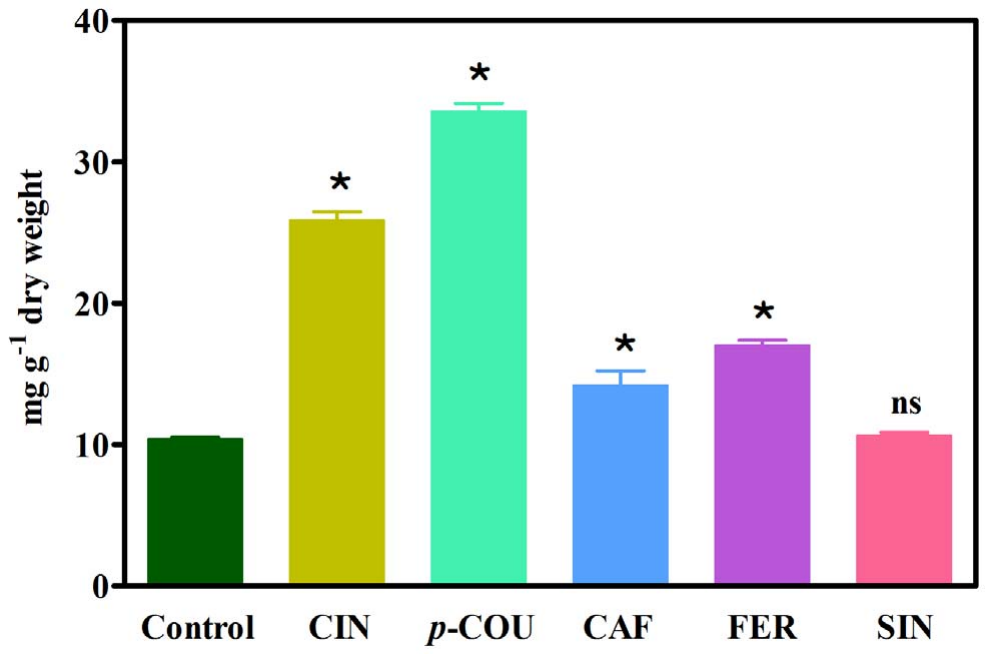
Lignin content in untreated (Control) soybean roots and roots treated with 1.0 mM cinnamic (CIN), *p*-coumaric (*p*-COU), caffeic (CAF), ferulic (FER) and sinapic (SIN) acids. Values (*N* = 4±SE) that are significantly different from the control (*P*≤0.05, Dunnett's multiple comparison test) are marked with an asterisk (*). ns = not significant.

The lignin contents of roots treated with cinnamic acid and its hydroxylated derivatives were significantly different from those of the control (Figure 1). The exposure of soybean roots to cinnamic, *p*- coumaric, caffeic and ferulic acids increased lignin content by 249%, 266%, 37% and 50%, respectively, compared with the control (10.4 mg g^−1^ dry weight).

### Effects of enzymatic inhibitors and allelochemicals on lignin monomer composition

#### Selective inhibitors

**Figure 2:**
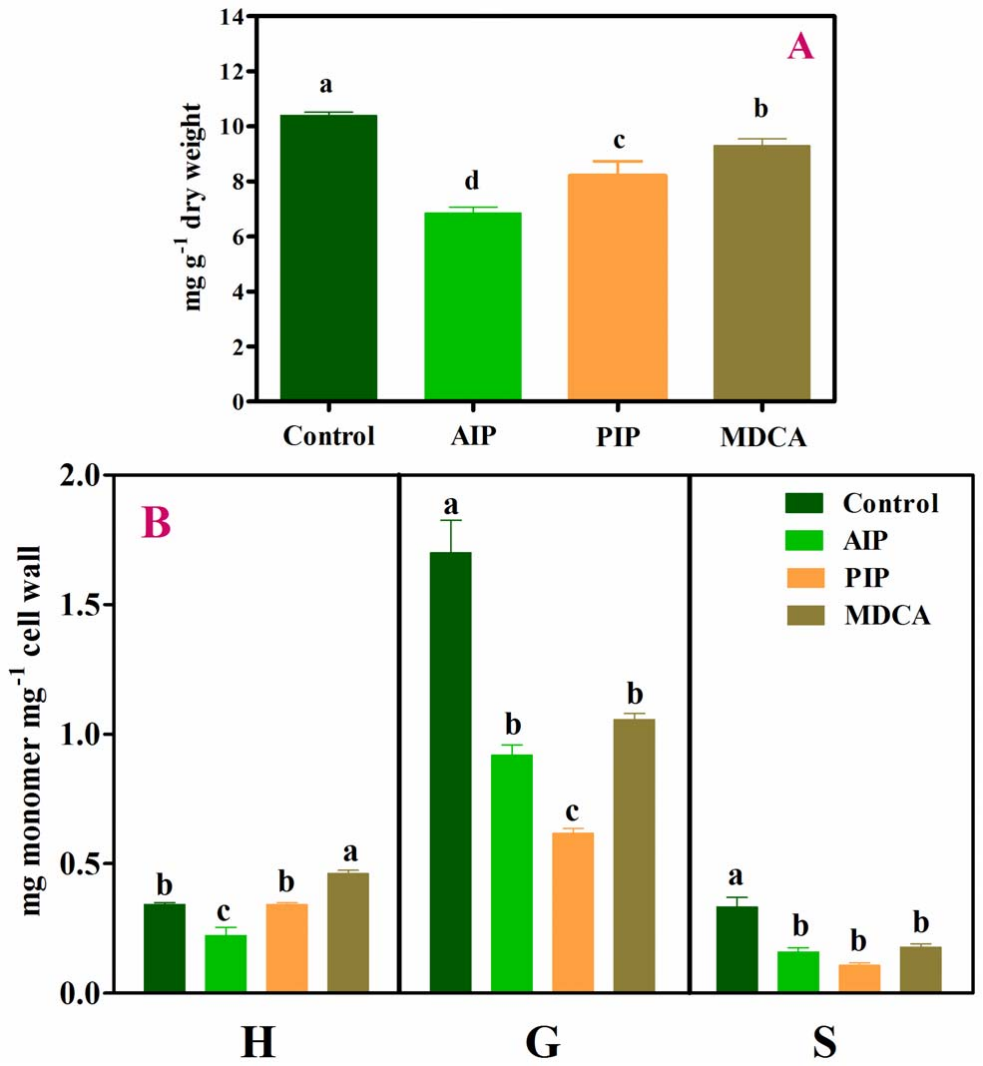
Lignin content (A) and lignin monomer composition (B) in untreated (Control) soybean roots and roots treated with 10 µM 2-aminoindan-2-phosphonic acid (AIP), 0.1 mM piperonylic acid (PIP) and 2.0 mM 3,4-(methylenedioxy)cinnamic acid (MDCA). Mean ± SE values (*N* = 4) followed by different letters are significantly different according to the Scott–Knott test (*P*≤0.05). H, *p*-hydroxyphenyl; G, guaiacyl; S, syringyl.

To confirm whether the enzyme inhibitors used in this work exert their effects on the phenylpropanoid pathway, thus affecting the production of lignin and its monomer composition, soybean seedlings were grown in the presence of these compounds (Figure 2). The results revealed that AIP, PIP and MDCA reduced lignin content by 33%, 20% and 10%, respectively, compared with the control (10.4 mg g^−1^ dry weight) (Figure 2A). Having already ascertained that lignin content was affected by these selective inhibitors, we investigated the lignin monomer composition by alkaline nitrobenzene oxidation (Figure 2B). This procedure degrades lignin, forming *p*-hydroxybenzaldehyde from *p*-hydroxyphenyl (H), vanillin from guaiacyl (G) and syringaldehyde from syringyl (S). Compared with their corresponding controls, AIP reduced the levels of H, G and S, and both PIP and MDCA reduced the G and S contents.

#### Cinnamic acid

**Figure 3:**
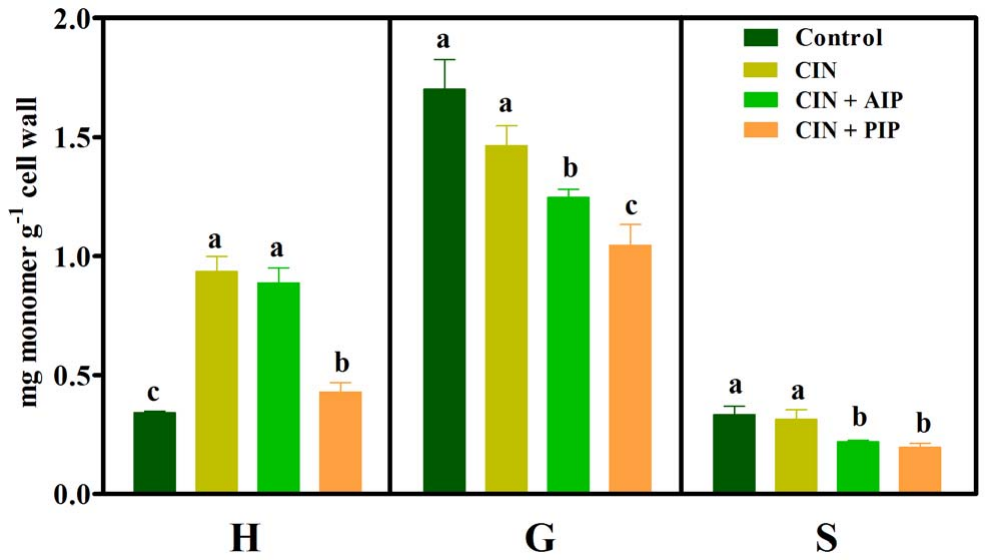
Lignin monomer composition in untreated (Control) soybean roots and roots treated with 1.0 mM cinnamic acid (CIN), 1.0 mM cinnamic acid plus 10 µM 2-aminoindan-2-phosphonic acid (CIN+AIP) and 1.0 mM cinnamic acid plus 0.1 mM piperonylic acid (CIN+PIP). Mean ± SE values (*N* = 4) followed by different letters are significantly different according to the Scott–Knott test (*P*≤0.05). H, *p*-hydroxyphenyl; G, guaiacyl; S, syringyl.

A relevant increase (174%) in the H lignin content was noted in roots exposed to cinnamic acid compared with the control (Figure 3). This increase reveals that the exposure of roots to cinnamic acid plus AIP (CIN+AIP) reduced G and S monomers compared with cinnamic acid (CIN) treatment alone. Additionally, treatment with CIN plus PIP reduced the contents of all monomers compared with the allelochemical alone.

#### p-Coumaric acid

**Figure 4:**
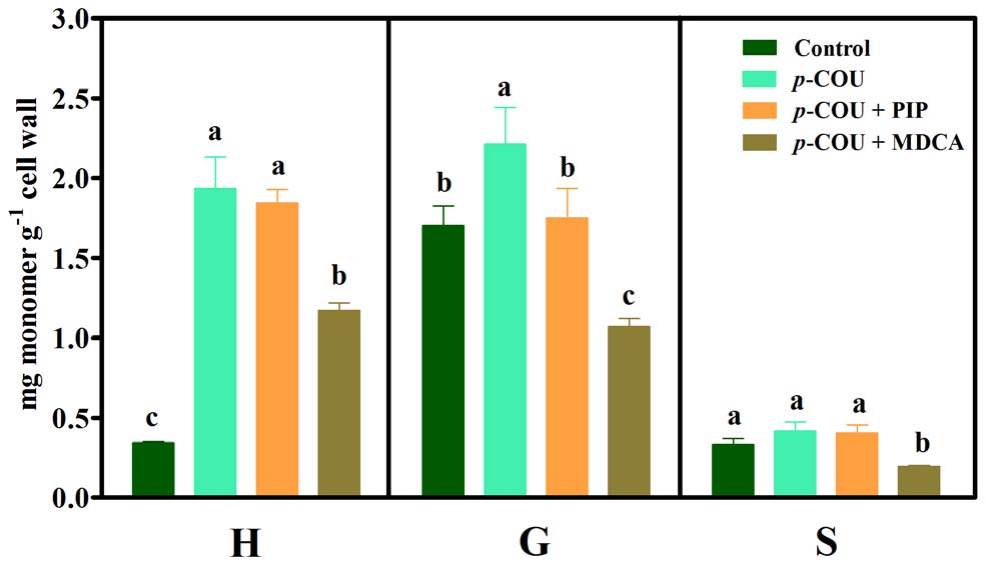
Lignin monomer composition in untreated (Control) soybean roots and roots treated with 1.0 mM *p*-coumaric acid (*p*-COU), 1.0 mM *p*-coumaric acid plus 0.1 mM piperonylic acid (*p*-COU+PIP) and 1.0 mM *p*-coumaric acid plus 2.0 mM 3,4-(methylenedioxy)cinnamic acid (*p*-COU+MDCA). Mean ± SE values (*N* = 4) followed by different letters are significantly different according to the Scott–Knott test (*P*≤0.05). H, *p*-hydroxyphenyl; G, guaiacyl; S, syringyl.

Compared with the corresponding controls, *p*-coumaric acid significantly increased H (430%) and G (30%) contents (Figure 4). The *p*-coumaric acid plus PIP (*p*-COU+PIP) treatment did not affect H and S contents and slightly reduced G compared with the allelochemical (*p*-COU) alone. On the other hand, all monomers were significantly reduced after *p*-COU plus MDCA treatment compared with *p*-coumaric acid treatment.

#### Caffeic acid

**Figure 5:**
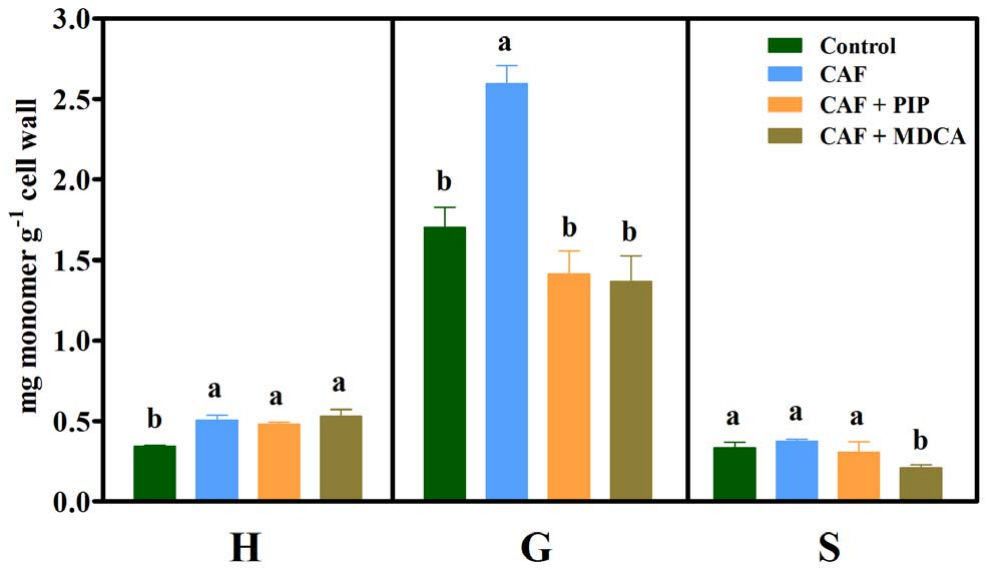
Lignin monomer composition in untreated (Control) soybean roots and roots treated with 1.0 mM caffeic acid (CAF), 1.0 mM caffeic acid plus 0.1 mM piperonylic acid (CAF+PIP) and 1.0 mM caffeic acid plus 2.0 mM 3,4-(methylenedioxy)cinnamic acid (CAF+MDCA). Mean ± SE values (*N* = 4) followed by different letters are significantly different according to the Scott–Knott test (*P*≤0.05). H, *p*-hydroxyphenyl; G, guaiacyl; S, syringyl.

Following treatment with caffeic acid, the H and G monomer contents increased by 47% and 52%, respectively, compared with the control (Figure 5). The exposure of roots to the allelochemical plus inhibitor (CAF+PIP) significantly reduced G content compared with caffeic acid (CAF) treatment alone. In conjunction with caffeic acid, MDCA reduced the content of G and S monomers compared with the allelochemical treatment.

#### Ferulic acid

**Figure 6:**
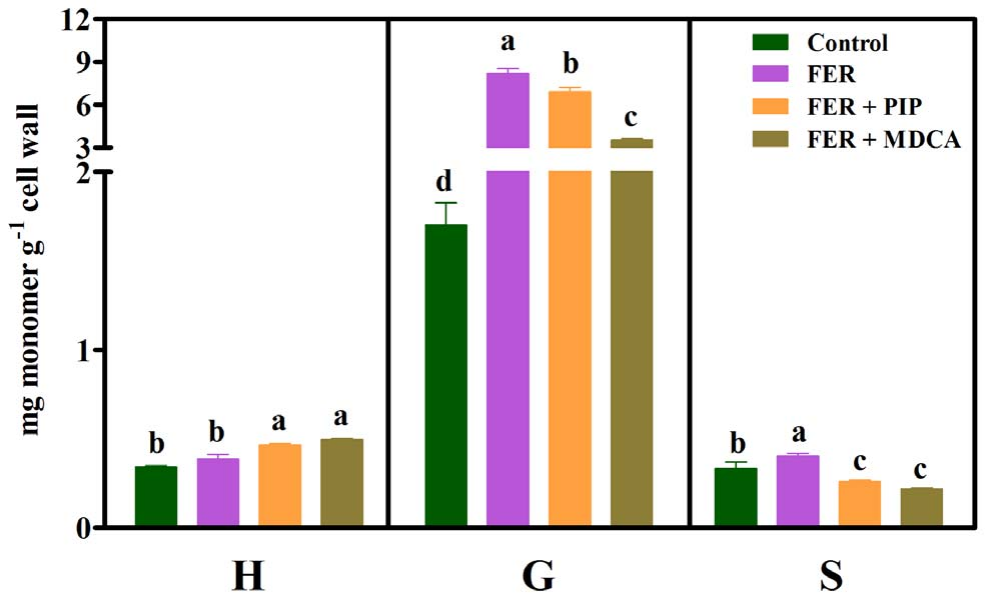
Lignin monomer composition in untreated (Control) soybean roots and roots treated with 1.0 mM ferulic acid (FER), 1.0 mM ferulic acid plus 0.1 mM piperonylic acid (FER+PIP) and 1.0 mM ferulic acid plus 2.0 mM 3,4-(methylenedioxy)cinnamic acid (FER+MDCA). Mean ± SE values (*N* = 4) followed by different letters are significantly different according to the Scott–Knott test (*P*≤0.05). H, *p*-hydroxyphenyl; G, guaiacyl; S, syringyl.

The analysis of monomer contents (Figure 6) revealed significant increases in the contents of G (380%) and S (21%) after exposure to ferulic acid compared with the corresponding controls. The treatments with ferulic acid plus PIP (FER+PIP) or ferulic acid plus MDCA (FER+MDCA) significantly reduced G and S contents compared with their levels in the allelochemical-treated roots.

#### Sinapic acid

**Figure 7:**
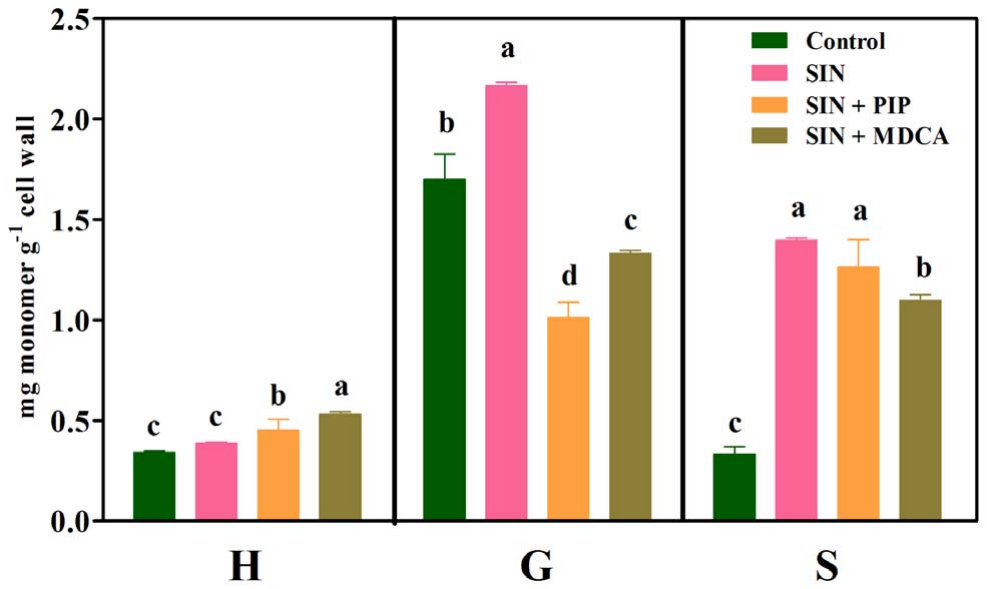
Lignin monomer composition in untreated (Control) soybean roots and roots treated with 1.0 mM sinapic acid (SIN), 1.0 mM sinapic acid plus 0.1 mM piperonylic acid (SIN+PIP) and 1.0 mM sinapic acid plus 2.0 mM 3,4-(methylenedioxy)cinnamic acid (SIN+MDCA). Mean ± SE values (*N* = 4) followed by different letters are significantly different according to the Scott–Knott test (*P*≤0.05). H, *p*-hydroxyphenyl; G, guaiacyl; S, syringyl.

Sinapic acid increased the contents of G (27%) and S (318%) compared with the corresponding controls (Figure 7). The sinapic acid plus PIP (SIN+PIP) treatment significantly reduced the G monomer content, whereas the sinapic acid plus MDCA (SIN+MDCA) treatment reduced the G and S contents compared with their levels in the allelochemical-treated roots.

#### H+G+S content and H∶G∶S ratio

Table 2 summarizes the results of lignin (referred to as the sum of H+G+S) measurements. The allelochemicals increased the H+G+S contents by 14% (cinnamic acid), 92% (*p*-coumaric acid), 46% (caffeic acid), 278% (ferulic acid) and 66% (sinapic acid) compared with the control. The same table reveals that the H∶G∶S ratio was altered by the action of allelochemicals as follows: Cinnamic and *p-*coumaric acids increased the H form, caffeic acid slightly increased the G form, ferulic acid increased the G unit and sinapic acid increased the S monomer.

**Table 2:** Monomer (H+G+S) composition and H∶G∶S ratio in soybean seedlings treated for 24 h with 1.0 mM cinnamic (CIN), *p*-coumaric (*p*-COU), caffeic (CAF), ferulic (FER) and sinapic (SIN) acids.

	Control	CIN	*p*-COU	CAF	FER	SIN
**H+G+S**	2.37±0.09	2.71±0.16^*^	4.57±0.44^*^	3.47±0.09^*^	8.98±0.38^*^	3.95±0.02^*^
**H∶G∶S**	14∶72∶14	34∶54∶12	42∶48∶10	15∶75∶10	4∶91∶5	10∶55∶35

The results are expressed in mg monomer g^−1^ cell wall.

H, *p*-hydroxyphenyl; G, guaiacyl; S, syringyl. Values (*N* = 4 ± SE) that are significantly different from the control (*P*≤0.05, Dunnett's multiple comparison test) are marked with an asterisk (*).

## Discussion

The susceptibility of different plant species to allelochemicals is widely known, and root growth is one of the main processes affected by these compounds [1]. The experimental conditions established in this study were chosen because root growth is characterized by high metabolic activity, and lignification begins in the early stages of seedling growth [18]. Cinnamic acid and hydroxylated derivatives such as *p*-coumaric, caffeic, ferulic and sinapic acids inhibit the root length and the fresh and dry weights of many plant species, including canola [15], [19], mung bean [16], pea [20], *Arabidopsis thaliana* [21] and cucumber [22], among others [23]. In the current research, we also found that these compounds (with the exception of sinapic acid) reduced the root growth and the fresh and dry weights of soybean seedlings (Table 1). These findings confirm the susceptibility of soybean to these allelochemicals and reinforce the role of phenylpropanoid derivatives as allelopathic agents [23].

An important fact observed in the present study was that soybean root growth decreased after exposure to most of the allelochemicals (Table 1), and this was followed by enhanced lignin production (Figure 1) and changes in lignin monomer composition (Figure 3 to 7). Previous results revealed that the reduced growth of soybean roots caused by ferulic [9], [14], *p*-coumaric [12] and caffeic [13] acids was associated with an effective performance of the phenylpropanoid pathway and premature cell wall lignification. So, a direct influx of these allelochemicals into the phenylpropanoid pathway followed by increases in the contents of lignin monomers, which solidify the cell wall and restrict root growth, has been postulated as a theoretical mode of action. Herein, we aim to confirm this hypothesis.

**Figure 8:**
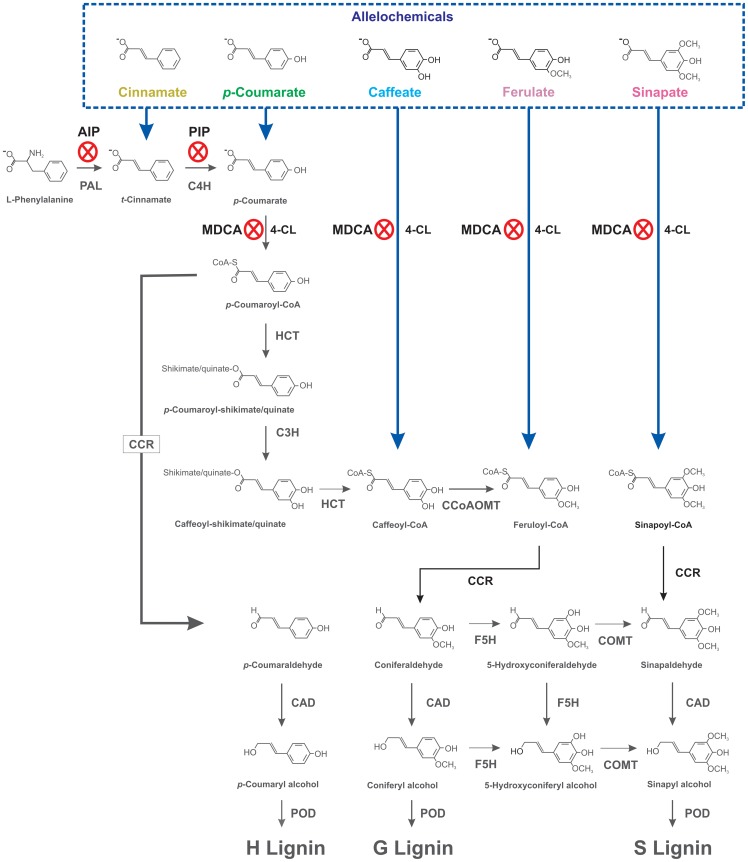
A simplified scheme for the synthesis of lignin and the proposed mode of action for cinnamic acid and its hydroxylated derivatives in soybean roots. PAL, phenylalanine ammonia-lyase; C4H, cinnamate 4-hydroxylase; 4CL, 4-coumarate:CoA ligase; HCT, *p*-hydroxycinnamoyl-CoA:shikimate/quinate *p*-hydroxycinnamoyl transferase; C3H, *p*-coumarate 3-hydroxylase; CCoAOMT, caffeoyl-CoA 3-*o*-methyltransferase; CCR, cinnamoyl-CoA reductase; CAD, cinnamyl alcohol dehydrogenase; F5H, ferulate 5-hydroxylase; COMT, caffeic acid 3-*o*-methyltransferase; POD, peroxidase. H, *p*-hydroxyphenyl; G, guaiacyl; S, syringyl. AIP, PIP and MDCA are inhibitors of PAL, C4H and 4CL, respectively.

To verify whether the exogenously applied allelochemicals are channeled via specific entry points of the phenylpropanoid pathway, we performed experiments using three selective enzyme inhibitors: AIP [24], PIP and MDCA [25]. AIP is a competitive inhibitor of PAL, PIP is an irreversible inhibitor of C4H and MCDA is a competitive inhibitor of 4CL (Figure 8). As expected, the exposure of soybean roots to these enzymatic inhibitors reduced the total lignin content (Figure 2A) and the amount of lignin monomers (Figure 2B). Subsequent experiments revealed that the exposure of soybean roots to a mixture of cinnamic acid plus a PAL inhibitor (CIN+AIP) did not affect the amount of H monomer compared with the allelochemical alone (Figure 3). It is interesting to note that this monomeric form was significantly increased by cinnamic acid alone. The results with CIN+AIP indicate that the PAL inhibitor did not prevent either the influx of the allelochemical into the metabolic pathway or the formation of H. In agreement with the results obtained with cinnamic acid alone, the sum of H+G+S (Table 2) increased and the H∶G∶S ratio (34∶54∶12) was altered (especially with respect to the H form) compared with the control ratio (14∶72∶14). In contrast, the mixture of cinnamic acid plus an inhibitor of C4H (CIN+PIP) reduced the amount of H, G and S monomers compared with allelochemical exposure. In brief, these findings indicate that exogenously applied cinnamic acid enters, via the C4H reaction, into the phenylpropanoid pathway and enhances the production of the H monomer (Figure 8).

The results obtained with *p*-coumaric acid showed significant increases in the H and G monomers compared with the control (Figure 4), and even greater increases have been confirmed (at least for the H unit) after treatment with *p*-coumaric acid plus PIP (*p*-COU+PIP). This is in agreement with the fact that PIP acts before the entry point of *p*-coumaric acid in the pathway [26]. The contents of all monomers were reduced after *p*-coumaric acid plus MDCA (*p*-COU+MDCA) treatment compared with allelochemical treatment alone. Because MDCA affects the 4CL reaction, the exogenous *p*-coumaric acid was blocked from accessing the phenylpropanoid pathway at this metabolic point [26] (Figure 8). Additionally, the sum of H+G+S increased after allelochemical treatment with significant changes in the H form (Table 2). These results strengthen the findings of Zanardo et al. [12], which suggest that exogenously applied *p*-coumaric acid can be channeled, via the 4CL reaction, into the phenylpropanoid pathway. This influx increases the contents of H and G monomers (Figure 4) associated with a decrease in root growth.

The H and especially G monomers increased under the action of caffeic acid (Figure 5). Caffeic acid plus PIP (CAF+PIP) or MDCA (CAF+MDCA) reduced the content of G monomer compared with the allelochemical alone. When compared with the control, the slight caffeic acid-induced increase in H monomer content suggests a possible redistribution of the metabolites and the monomers in this complex metabolic network. This is plausible because the H∶G∶S ratio was not altered compared to the control (Table 2). Regardless, the findings obtained after treatment with caffeic acid plus MDCA indicate that this allelochemical can be channeled, via the 4CL reaction, into the phenylpropanoid pathway followed by an enhanced lignin content and reduced soybean root growth as suggested by Bubna et al. [13].

The exposure of soybean seedlings to ferulic acid increased the contents of lignin (Figure 1) and the G and S monomers (Figure 6), thus changing the H+G+S and the H∶G∶S ratio (Table 2) and reducing the root growth (Table 1). G and S monomer contents were reduced after treatment with ferulic acid plus MDCA (FER+MDCA) compared with allelochemical treatment alone (Figure 6). Because MDCA inhibits 4CL, the influx of ferulic acid into the phenylpropanoid pathway was blocked at this entry point (Figure 8). These findings, together with those obtained by dos Santos et al. [14], strengthen the hypothesis that ferulic acid, like *p*-coumaric and caffeic acids, can be channeled into the phenylpropanoid pathway via the 4CL reaction, and previous work supports this hypothesis. Hamada et al. [27] reported that exogenous ferulic acid was converted to feruloyl and subsequently to coniferyl and sinapyl alcohols in poplar (*Populus alba*) callus. Additionally, labeled ferulic acid was incorporated into guaiacyl (G) and syringyl (S) lignin followed by an increase in cell wall lignification in robinia (*Robinia pseudoacacia*) [28].

Finally, the results showed that sinapic acid (at least for 1.0 mM) did not affect soybean root growth (Table 1) or total lignin (Figure 1). However, this compound increased the contents of G and, more significantly, S monomers (Figure 7). There is no convincing explanation for the findings obtained after the treatments with sinapic acid plus PIP (SIN+PIP) or MDCA (SIN+MDCA) because the G monomer level was the most reduced, in contrast with that of the S unit. Nevertheless, the sum of H+G+S increased (Table 2) and the H∶G∶S ratio (10∶55∶35) was altered (especially with respect to the S monomer) compared with the control (14∶72∶14). This result suggests that exogenously applied sinapic acid, like other allelochemicals used in this work, can be channeled into the phenylpropanoid pathway. Previous research indicating that sinapic acid can be esterified in the cell wall supports this finding [29].

The most relevant results obtained in the present research indicate that 1) cinnamic, *p*-coumaric, caffeic and ferulic acids reduce root growth and increase lignin content; 2) the content of H monomer was considerably increased after the roots were treated with cinnamic and *p*-coumaric acids; 3) the levels of G monomer increased after treatment with *p*-coumaric, caffeic and ferulic acids; 4) the S content increased in roots treated with sinapic acid; and 5) the use of selective enzymatic inhibitors indicated the specific entry points of allelochemicals into the phenylpropanoid pathway, influencing the production of lignin and its monomer composition. These experimental findings have enabled us to provide a working model for the role of cinnamic acid and its hydroxylated derivatives as allelochemicals (Figure 8). In this model, exogenously applied compounds are channeled into the phenylpropanoid pathway and subsequently converted into their corresponding monolignols by the sequential action of 4CL, CCR and CAD, which generate the H, G and S units of the lignin polymer. In conclusion, these results confirm our hypothesis that allelochemical-induced inhibition of soybean root growth may be due to the excessive production of lignin, which solidifies the cell wall and restricts plant growth.

## References

[1] Weir TL, Park S-W, Vivanco JM (2004) Biochemical and physiological mechanisms mediated by allelochemicals. Current Opinion in Plant Biology 7: 472–479. 15231272 10.1016/j.pbi.2004.05.007

[2] Macias FA (1995) Allelopathy in the search for natural herbicides model. In: Inderjit, Dakshini KMM, Enhellig FA, eds. Allelopathy, current status and future goals. Washington: American Chemical Society, 311–329.

[3] Siqueira JO, Nair MG, Hammerschmidt R, Safir GR, Putnam AR (1991) Significance of phenolic compounds in plant-soil microbial systems. Critical Reviews in Plant Sciences 10: 63–121.

[4] Vanholme R, Morreel K, Darrah C, Oyarce P, Grabber JH, et al (2012) Metabolic engineering of novel lignin in biomass crops. New Phytologist 196: 978–1000. 23035778 10.1111/j.1469-8137.2012.04337.x

[5] Moura JC, Bonine CA, de Oliveira FVJ, Dornelas MC, Mazzafera P (2010) Abiotic and biotic stresses and changes in the lignin content and composition in plants. Journal of Integrative Plant Biology 52: 360–376. 20377698 10.1111/j.1744-7909.2010.00892.x

[6] Cabane M, Afif D, Hawkins S (2012) Lignins and abiotic stresses. In: Jouann L, Lapierre C, eds. Lignins: Biosynthesis, Biodegradation and Bioengineering. San Diego: Elsevier Academic Press, 219–262.

[7] Politycka B (1999) Ethylene-dependent activity of phenylalanine ammonia-lyase and lignin formation in cucumber roots exposed to phenolic allelochemicals. Acta Societatis Botanicorum Poloniae 68: 123–127.

[8] Devi SR, Prasad MNV (1996) Ferulic acid mediated changes in oxidative enzymes of maize seedlings: implications in growth. Biologia Plantarum 38: 387–395.

[9] dos Santos WD, Ferrarese MLL, Finger A, Teixeira ACN, Ferrarese-Filho O (2004) Lignification and related enzymes in *Glycine max* root growth-inhibition by ferulic acid. Journal of Chemical Ecology 30: 1199–1208. 10.1023/b:joec.0000030272.83794.f0 15303323

[10] Böhm PAF, Zanardo FML, Ferrarese MLL, Ferrarese-Filho O (2006) Peroxidase activity and lignification in soybean root growth-inhibition by juglone. Biologia Plantarum 50: 315–317.

[11] Soares AR, Ferrarese MLL, Siqueira-Soares RC, Marchiosi R, Finger-Teixeira A, et al (2011) The allelochemical L-DOPA increases melanin production and reduces reactive oxygen species in soybean roots. Journal of Chemical Ecology 37: 891–898. 21710366 10.1007/s10886-011-9988-2

[12] Zanardo DIL, Lima RB, Ferrarese MLL, Bubna GA, Ferrarese-Filho O (2009) Soybean root growth inhibition and lignification induced by *p*-coumaric acid. Environmental and Experimental Botany 66: 25–30.

[13] Bubna GA, Lima RB, Zanardo DYL, dos Santos WD, Ferrarese MLL, et al (2011) Exogenous caffeic acid inhibits the growth and enhances the lignification of the roots of soybean (*Glycine max*). Journal of Plant Physiology 168: 1627–1633. 21489652 10.1016/j.jplph.2011.03.005

[14] dos Santos WD, Ferrarese MLL, Nakamura CV, Mourão KSM, Mangolin CA, et al (2008) Soybean (*Glycine max*) root lignification induced by ferulic acid. The possible mode of action. Journal of Chemical Ecology 34: 1230–1241. 18626717 10.1007/s10886-008-9522-3

[15] Ng PLL, Ferrarese MLL, Huber DA, Ravagnani ALS, Ferrarese-Filho O (2003) Canola (*Brassica napus* L.) seed germination influenced by cinnamic and benzoic acids and derivatives: effects on peroxidase. Seed Science and Technology 31: 39–46.

[16] Batish DR, Singh HP, Kaur S, Kohli RK, Yadav SS (2008) Caffeic acid affects early growth, and morphogenetic response of hypocotyl cuttings of mung bean (*Phaseolus aureus*). Journal of Plant Physiology 165: 297–305. 17643552 10.1016/j.jplph.2007.05.003

[17] Singh HP, Kaur S, Batish DR, Kohli KR (2009) Caffeic acid inhibits *in vitro* rooting in mung bean [*Vigna radiata* (L.) Wilczek] hypocotyls by inducing oxidative stress. Plant Growth Regulation 57: 21–30.

[18] Donaldson LA (2001) Lignification and lignin topochemisty - an ultrastructural view. Phytochemisty 57: 859–873. 10.1016/s0031-9422(01)00049-8 11423137

[19] Baleroni CRS, Ferrarese MLL, Braccini AL, Scapim CA, Ferrarese-Filho O (2000) Effects of ferulic and caffeic acids on canola (*Brassica napus* L. cv. Hyola 401) seed germination. Seed Science and Technology 28: 201–207.

[20] Vaughan D, Ord B (1990) Influence of phenolic acids on morphological changes in roots of *Pisum sativum* . Journal of Science Food and Agriculture 52: 289–299.

[21] Reigosa MJ, Pazos-Malvido E (2007) Phytotoxic effects of 21 plant secondary metabolites on A*rabidopsis thaliana* germination and root growth. Journal of Chemical Ecology 33: 1456–1466. 17577597 10.1007/s10886-007-9318-x

[22] Politycka B, Mielcarz B (2007) Involvement of ethylene in growth inhibition of cucumber root by ferulic and *p*-coumaric acids. Allelopathy 19: 451–460.

[23] Einhellig FA (1995) Characterization of the mechanisms of allelopathy. Modeling and experimental approaches. In: Cheng HH, Inderjit, Dakshini KMM. Allelopathy, Organisms, Processes and Applications. Washington: American Chemical Society. pp. 132–141.

[24] Appert C, Zon J, Amrhein N (2003) Kinetic analysis of the inhibition of phenylalanine ammonia-lyase by 2-aminoindan-2-phosphonic acid and other phenylalanine analogues. Phytochemistry 62: 415–422. 12620354 10.1016/s0031-9422(02)00561-7

[25] Chakraborty M, Karun A, Mitra A (2009) Accumulation of phenylpropanoid derivatives in chitosan-induced cell suspension culture of *Cocos nucifera* . Journal of Plant Physiology 166: 63–71. 18448193 10.1016/j.jplph.2008.02.004

[26] Schoch GA (2002) Chemical inactivation of the cinnamate 4-hydroxylase allows for the accumulation of salicylic acid in elicited cells. Plant Physiology 130: 1022–1031. 12376665 10.1104/pp.004309 PMC166627

[27] Hamada K, Tsutsumi Y, Nishida T (2003) Treatment of poplar callus with ferulic and sinapic acids II: effects on related monolignol biosynthetic enzyme activities. Journal of Wood Science 49: 366–370.

[28] Yamauchi K, Fukushima K (2004) The regulation from guaiacyl to syringyl lignin in the differentiating xylem of *Robinia pseudoacacia* . Comptes Rendus Biologies 327: 791–797. 15587070 10.1016/j.crvi.2004.07.012

[29] Grabber JH (2005) How do lignin composition, structure, and cross-linking affect degradability? A review of cell wall model studies. Crop Science 45: 820–831.

